# Cutaneous Cholangiocarcinoma: An Interesting Presentation

**DOI:** 10.1097/GOX.0000000000002980

**Published:** 2020-08-19

**Authors:** Gregory Greco, Paul Jennings, Alexis Coleman, Syed Hussain Abbas

**Affiliations:** From the * Department of General Surgery, RWJ Barnabas Health, Monmouth Medical Center, Long Branch, N.J.; † Plastic, Cosmetic and Reconstructive Surgery, Red Bank, N.J.; ‡ Department of Pathology, RWJ Barnabas Health, Saint Barnabas Medical Center, Livingston, N.J.

## Abstract

This is a case of a 64-year-old white man with a history of CCA, originally diagnosed in May 2018 and returning in November 2019 with growing cutaneous nodules. These were removed for cosmetic and functional purposes. Pathologic findings of the lesions showed likely metastatic disease from his original CCA. This represents a relatively rare presentation of metastatic disease in the setting of CCA. In cases of CCA with metastatic spread, treatment is not curative and should be focused on measures to improve the patient’s quality of life. This includes acceptable cosmesis, as well as factors aiding in completing activities of daily living.

Intrahepatic cholangiocarcinoma (iCCA) is a relatively rare malignancy in the United States. According to data from the National Cancer Institute Surveillance, Epidemiology, and End Results program, United States comprise 15% of newly diagnosed primary liver malignancies annually, roughly 6300 cases per year.^1^ Presentation of the malignancy usually relates to biliary obstruction; however, signs and symptoms of the disease can rarely relate to metastatic disease as well.

Skin metastasis is rare, involving 0.7%–9% of all cancer patients.^2^ Lung and breast cancer are the leading causes of cutaneous metastasis in men and women, respectively.^2^

We present the case of a 64-year-old man with metastatic cholangiocarcinoma. He developed 2 cutaneous, firm, mobile, rapidly growing masses at the angle of the left mandible and in the left axilla. Excision was performed for both diagnostic and aesthetic purposes. The pathologic findings confirmed poorly differentiated carcinoma, whose morphology is similar to that of tissue samples removed from his original mass in the liver. This patient represents an interesting presentation of his disease, and his treatment highlights how surgical intervention in the setting of metastatic disease, while not curative, can be beneficial to the patient for improving quality of life.

## INTRODUCTION

Cholangiocarcinoma (CCA) refers to cancers arising from the biliary tree. Only 5%–10% of these are intrahepatic. About 19%–43% of iCCA are diagnosed incidentally, as patients may become symptomatic only with advancement of the disease, and may present only with vague abdominal symptoms.^3^ These tumors occur more frequently in Asia than in Western countries. Risk factors include primary sclerosing cholangitis, choledochal cysts, parasitic infections, hepatitis B and C, HIV, Lynch syndrome, and exposure to cigarette smoke. Cholangiocarcinoma most commonly metastasizes to other locations in the liver, regional lymph nodes, or the lung; however, metastasis to the bone have been described in rare cases.^4^ Curative treatment may only be obtained through surgical resection; however, even in patients with an R0 resection, recurrence rates remain high, between 49% and 64%.^3^ Chemotherapy in unresectable disease may be used for palliative measures.

## CASE DESCRIPTION

The patient is a 64-year-old white man with a history of cholangiocarcinoma, originally diagnosed in May 2018 after he presented with painless jaundice. Over 1 year after diagnosis and subsequent treatment, he began complaining of nodular lesions that had appeared near the left mandible and in the left axilla. These lesions had grown over the course of several weeks and had become bothersome. The lesion on his mandible made it difficult for the patient to shave. He was referred to a plastic surgeon for diagnosis and resection.

During his 2018 workup, imaging showed a 3.8 × 3.2 cm ill-defined, irregular heterogenous lesion in the left lobe of the liver. He underwent an esophagogastroduodenoscopy with endoscopic ultrasound and fine-needle aspiration of the lesion, revealing a poorly differentiated carcinoma with anaplastic features. He underwent endoscopic retrograde cholangio-pancreatography and cholangiogram that demonstrated a normal appearing common bile duct and common hepatic duct with an area of stricture extending from above the hilum to the bilateral intrahepatic ducts. He has had multiple stents placed in the intrahepatic bile ducts to relieve obstructive symptoms. He has been receiving chemotherapy under the direction of a medical oncologist; however, his lesions failed to resolve and appear to have seemingly spread, leading to his current presentation.

**Fig. 1.**
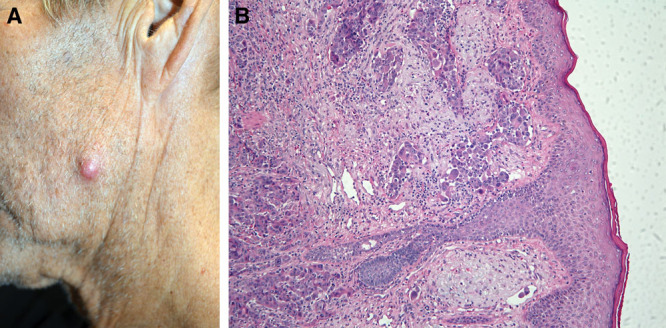
Facial cutaneous metastasis. A, 1.5 × 1.2 cm^2^ nodular left mandibular mass, gross appearance. B, Microscopic view showing poorly differentiated adenocarcinoma.

**Fig. 2.**
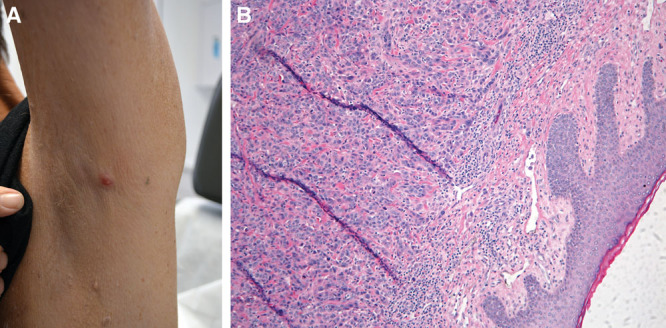
Axillary cutaneous metastasis. A, 1.0 cm left axillary mass, gross macroscopic view. B, Microscopic view.

He was taken for excision of his lesions in November 2019, 18 months after his original diagnosis. He was noted to have a 1.5 × 1.2 cm^2^ lesion at the angle of the left mandible (Fig. 1A) and a 1.0 cm lesion in the left axilla (Fig. 2A). These had a hard, fibrous consistency, suspicious for metastatic disease. Samples were sent for pathologic examination, which confirmed poorly differentiated carcinoma (Fig. 1B and 2B) with morphological characteristic similar to those of tissue samples removed from his original mass in the liver.

## DISCUSSION

Cholangiocarcinoma is a rare malignancy that generally presents with symptoms relating to biliary obstruction.^5^ Metastatic spread typically affects local lymphatics and other locations in the liver. Spread to bone and soft tissue has been described in literature, usually as individual case reports or low-power case series. Data on the frequency of spread to bones or soft tissues remain limited, but the consensus appears to be that overall prognosis is dismal.

Dowsiriroj et al^6^ reported 55 cases of spinal metastases from CCA, with a median survival of only 4.0 months. Chindaprasirt et al^4^ documented 5 cases of metastases to the appendicular skeleton, most commonly the humerus, and 1 case of metastasis to the scapula, with survival ranging from 4 weeks to 1 year. Liu et al^7^ compiled a literature review of 30 patients with cutaneous metastases, most commonly to the scalp, also with a median survival of 4.0 months.

In conclusion, cholangiocarcinoma has been shown to metastasize to bone and soft tissue. However, spread to these sites is rare among cases of metastatic CCA, as well as compared with other cancers known to metastasize to these sites, such as breast or lung cancer. Survival after such distant spread is universally poor; however, it should be noted that even among primary cases of CCA, 5-year survival rates only approach 30%, even with surgical resection.^3^ Preferred treatment for CCA focuses on surgical resection or liver transplant. When not possible, such as in metastatic disease, gemcitabine/cisplatin combination therapy has shown a survival benefit in CCA patients. Palliative options such as radiofrequency ablation, transarterial chemoembolization, drug-eluting bead-transarterial chemoembolization, selective intra-arterial radiotherapy, or external beam radiation therapy have been attempted,^3^ albeit with limited data on their results.
